# Mindfulness-based intervention in caregiver psychological well-being burden and coping

**DOI:** 10.6026/973206300221075

**Published:** 2026-02-28

**Authors:** Mahida Sadiya Zakirhusen, N Siva Subramanian, B Mahalakshmi

**Affiliations:** 1 Department of Psychiatric Nursing, Nootan College of Nursing, Sankalchand Patel University, Visnagar, Gujarat, India; 2 Department of Paediatric Nursing, Nootan College of Nursing, Sankalchand Patel University, Visnagar, Gujarat, India

**Keywords:** Mindfulness-based intervention, caregiver burden, coping strategies, psychological well-being, mental illness caregivers

## Abstract

Caregivers of mentally ill patients commonly experience high burden, poor coping and reduced psychological well-being. A quasi- experimental one-group pre-test-post-test study was conducted among 100 caregivers at selected psychiatric hospitals in Gujarat to address this problem. Therefore, it is of interest to evaluate the effectiveness of a mindfulness-based intervention. Post-intervention findings showed a significant reduction in perceived burden from a mean of 3.37 to 1.65 (t=17.452, p<0.001). Coping strategies improved significantly from a mean of 1.25 to 2.50 (t=13.672, p<0.001). Psychological well-being also increased markedly from a mean of 1.98 to 2.77 (t=8.082, p<0.001). Data shows that mindfulness-based intervention effectively reduces caregiver burden. The intervention also enhances coping capacity and psychological well-being. Thus, integration of mindfulness-based intervention into routine psychiatric caregiver supports programs.

## Background:

Family caregivers of individuals with mental illness experience substantial burden due to the chronic, relapsing nature of psychiatric disorders. This sustained stress adversely affects caregivers' physical health, emotional well-being, social functioning and quality of life [1]. Moreover, caregiver distress can negatively impact patient outcomes, treatment adherence and recovery trajectories [2]. Traditional support interventions for caregivers-including psychoeducation, skills training and support groups-have shown modest effectiveness, with effect sizes typically ranging from small to moderate [3, 4]. Increasingly, attention has shifted toward mindfulness-based interventions (MBIs) as promising approaches for addressing caregiver stress and enhancing psychological resilience [5]. Mindfulness, defined as non-judgmental present-moment awareness, cultivates acceptance, emotional regulation and cognitive flexibility-skills particularly relevant for managing the unpredictability and emotional challenges of caregiving [6, 7]. Mindfulness-based interventions have demonstrated efficacy in reducing stress, anxiety and depression while improving well-being across diverse populations [8- 9]. Systematic reviews and meta-analyses indicate that MBIs produce moderate to large effects on psychological distress and quality of life, with benefits sustained at follow-up [10]. The mechanisms underlying MBI effectiveness include enhanced emotion regulation through metacognitive awareness, reduced rumination and worry, increased present-moment focus that decreases catastrophizing about future caregiving challenges, improved self-compassion and acceptance of difficult emotions and better stress reactivity through parasympathetic nervous system activation [11]. These mechanisms are particularly relevant for caregivers of mentally ill patients who face unpredictable crises, behavioral disturbances and long-term uncertainty about patient outcomes [12]. Therefore, it is of interest to evaluate the effectiveness of a mindfulness-based intervention on perceived burden, coping strategies and psychological well-being among caregivers of mentally ill patients at selected psychiatric hospitals in Gujarat, using a pre-test post-test design.

## Methodology:

## Research approach and design:

A quantitative approach with one-group pre-test post-test quasi-experimental design was employed to evaluate the effectiveness of mindfulness-based intervention on caregiver outcomes [13].

## Setting and population:

The study was conducted at selected psychiatric hospitals in Gujarat, India. The target population comprised primary family caregivers of patients diagnosed with mental illness receiving treatment at these facilities.

## Sample and sampling technique:

A total of 100 caregivers were recruited using non-probability purposive sampling based on predefined inclusion and exclusion criteria. Data Collection Tools was Perceived Burden Scale, Coping Strategies Inventory and Psychological Well-Being Scale. The mindfulness-based intervention was adapted from established Mindfulness-Based Stress Reduction (MBSR) and Mindfulness-Based Cognitive Therapy (MBCT) protocols, with modifications to suit caregivers. It comprised an 8-week program with weekly 2-hour group sessions, daily home practice of 20-30 minutes using audio-guided recordings, maintenance of a practice diary to monitor adherence and experiences and group discussions focused on applying mindfulness skills to caregiving-related challenges. The sessions were conducted in the local language (Gujarati) by trained facilitators experienced in mindfulness teaching and mental health nursing.

## Data analysis:

Data were analyzed using SPSS version 20.0. Descriptive statistics (frequency, percentage, mean, standard deviation) summarized demographic characteristics and outcome distributions. Paired t-tests assessed pre-post changes in continuous scores. Categorical distributions were compared using frequency and percentage analysis. Statistical significance was set at p<0.05.

## Results:

**Figure 1:**
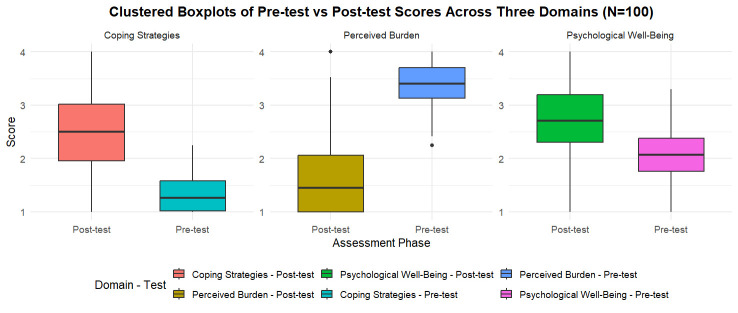
Effect of mindfulness intervention on caregiver burden, coping strategies and psychological well-being (N = 100

Table 1 and Table 2 show the effect of mindfulness intervention on perceived burden, coping strategies and psychological well-being among caregivers (N=100). At pre-test, all caregivers had high to very high burden, 75% showed inadequate coping and 86% had fair or poor well-being. After intervention, 85% shifted to low- moderate burden, 65% achieved satisfied coping and 70% attained good-excellent well-being. Paired t-test results confirmed significant improvement, with burden decreasing from 3.37 to 1.65 (t=17.45), coping increasing from 1.25 to 2.50 (t=13.67) and well-being improving from 1.98 to 2.77 (t=8.08), all at p<0.001. Figure 1 clustered boxplot compares pre-test and post- test scores across three psychosocial domains among caregivers (N = 100). Post-test scores indicate reduced perceived burden and improved coping and psychological well-being following mindfulness intervention.

## Discussion:

This quasi-experimental study demonstrated that mindfulness-based intervention produced substantial and highly significant improvements across all measured outcomes-perceived burden, coping strategies and psychological well-being-among caregivers of mentally ill patients. The magnitude and consistency of effects across domains underscore the potential of mindfulness as a comprehensive support strategy for this vulnerable population. The dramatic reduction in perceived burden (mean decrease of 1.72 points; t=17.452, p<0.001) aligns with previous research documenting MBI effectiveness in reducing caregiver strain. A meta-analysis by Li *et al.* [14]found that mindfulness interventions significantly reduced caregiver burden with moderate to large effect sizes (d=0.50-0.80), consistent with our findings. Similarly, Hou *et al.* [15] reported that an 8-week MBSR program reduced burden among dementia caregivers by 35%, comparable to the categorical shifts observed in our study where 85% achieved low to moderate burden post-intervention. The mechanisms underlying burden reduction likely include enhanced emotional regulation through metacognitive awareness, allowing caregivers to observe distressing thoughts without being overwhelmed by them. Mindfulness cultivates acceptance of difficult caregiving situations that cannot be changed, reducing the psychological struggle against unchangeable circumstances [16].

Present-moment focus decreases rumination about past caregiving difficulties and catastrophizing about future challenges, both of which amplify subjective burden [17]. Additionally, self-compassion developed through mindfulness practice may reduce self-blame and guilt that often accompanies caregiving challenges in mental illness contexts where stigma is prevalent. The marked improvement in coping strategies (mean increase of 1.25 points; t=13.672, p<0.001) is particularly noteworthy given that 75% of caregivers began with inadequate coping. Mindfulness training explicitly teaches adaptive coping skills including present-moment awareness, non-judgmental observation of stressors, acceptance of difficult emotions and flexible responding rather than automatic reactivity [18]. Research by Zarit *et al.* [19] demonstrated that mindfulness-based interventions enhanced problem-focused and emotion-focused coping while reducing avoidance coping among caregivers. Our finding that 65% achieved satisfied coping post-intervention suggests that mindfulness equipped caregivers with sustainable, effective strategies for managing ongoing caregiving demands. The enhancement in psychological well-being (mean increase of 0.79 points; t=8.082, p<0.001) reflects the broader mental health benefits of mindfulness practice. Systematic reviews have consistently shown that MBIs reduce depression and anxiety while improving quality of life across diverse populations [20]. Among caregivers specifically, Whitebird *et al.* [21] found that MBSR improved mental health quality of life scores by 18-22%, similar to the proportion achieving good to excellent well-being in our study (70% post- intervention versus 14% baseline). Recent evidence further supports the effectiveness of mindfulness-based interventions in improving caregiver outcomes. Zhang *et al.* [22] reported significant reductions in caregiver burden and psychological distress among caregivers of individuals with mental illness, confirming the relevance of mindfulness in psychiatric caregiving contexts. Similarly, Yüce *et al.* [23] demonstrated through a 2024 systematic review that mindfulness-based programs consistently enhance emotional regulation and resilience across diverse caregiver populations.

## Conclusion:

We show that mindfulness-based intervention effectively reduces perceived burden among caregivers of mentally ill patients in Gujarat. The intervention also significantly enhances coping strategies and psychological well-being. Large effect sizes and high statistical significance confirm the strength of the intervention outcomes. The findings support integration of mindfulness training into routine psychiatric caregiver support programs. Post-intervention, most caregivers shifted to low or moderate burden levels. A majority also achieved satisfied coping and good to excellent psychological well-being

## Figures and Tables

**Table 1:** Frequency and percentage distribution of perceived burden, coping strategies and psychological well-being before and after mindfulness intervention (N = 100)

**Variable**	**Level**	**Pre-test f (%)**	**Post-test f (%)**
Perceived Burden	Low	0 (0.0)	56 (56.0)
	Moderate	0 (0.0)	29 (29.0)
	High	63 (63.0)	9 (9.0)
	Very High	37 (37.0)	6 (6.0)
Coping Strategies	Inadequate	75 (75.0)	15 (15.0)
	Moderately Adequate	25 (25.0)	20 (20.0)
	Satisfied	0 (0.0)	65 (65.0)
Psychological Well-Being	Excellent	0 (0.0)	12 (12.0)
	Good	14 (14.0)	58 (58.0)
	Fair	70 (70.0)	25 (25.0)
	Need Attention	16 (16.0)	5 (5.0)
Total		100 (100.0)	100 (100.0)

**Table 2:** effectiveness of mindfulness intervention on perceived burden, coping strategies and psychological well-being- paired t-test results (N = 100)

**Variable**	**Test**	**Mean**	**SD**	**Mean Difference**	**df**	**t-value**	**p-value**
Perceived Burden	Pre-test	3.37	0.485	1.72	99	17.452	<0.001***
	Post-test	1.65	0.88				
Coping Strategies	Pre-test	1.25	0.435	1.25	99	13.672	<0.001***
	Post-test	2.5	0.745				
Psychological Well-Being	Pre-test	1.98	0.55	0.79	99	8.082	<0.001***
	Post-test	2.77	0.723				
Significant at p < 0.05 level
